# Ultrasound-Assisted Continence Care Support in an Inpatient Care Setting: Protocol for a Pilot Implementation Study

**DOI:** 10.2196/47025

**Published:** 2023-07-13

**Authors:** Sebastian Hofstetter, Madeleine Ritter-Herschbach, Dominik Behr, Patrick Jahn

**Affiliations:** 1 AG-Versorgungsforschung/Pflege im Krankenhaus Departement of Internel Medicine Martin-Luther-University Halle-Wittenberg Halle (Saale) Germany; 2 Dorothea-Erxleben-Learning Centre (Skills Lab) Medical Faculty Martin-Luther-University Halle-Wittenberg Halle (Saale) Germany; 3 Translationsregion digitale Gesundheitsversorgung Medical Faculty Martin-Luther-Universität Halle-Wittenberg Halle (Saale) Germany

**Keywords:** bladder control, bladder dysfunction, continence care, digitization, incontinence, nursing, selfmanagement, ultrasound, urology, user requirements

## Abstract

**Background:**

This nonrandomized exploratory intervention and feasibility study examines how digital assistive technology (DAT), comprising a DFree ultrasound sensor, affects nursing care for continence support and evaluates nurses’ willingness to incorporate DAT into the planning and practical implementation of care processes.

**Objective:**

The relief provided by DFree in the clinical care setting and the extent to which it supports nursing care for activities of daily living pertaining to “micturition” is unclear. DAT DFree is expected to reduce nurses’ workload in clinical continence-care settings and was designed as a human-technology interaction that ensures a high level of usability for the subjects (ie, the nurses) and increases user acceptance by at least one level (eg, from average to slightly above average) during the study.

**Methods:**

Approximately 45 nurses from neurology, neurosurgery, and geriatric medicine clinics and polyclinics at the University Medicine Halle will be included in the 90-day (3-month) intervention on-site in the respective wards. After the wards are equipped with digital technologies, the participating nurses will be trained to use DFree and will be able to select DFree as a possible patient-care resource if the anamnesis includes bladder dysfunction among only patients who are willing to participate. The willingness of nurse participants to use DFree in planning their care process will be assessed using the Technology Usage Inventory at 3 measurement points. The primary target values include the results of the multidimensional Technology Usage Inventory assessment that will be processed using descriptive statistics. Ten participating nurses will be invited to conduct extensive guided interviews that are intended to provide information about the device’s usefulness and feasibility in the specific field of continence care and possible improvements.

**Results:**

It is expected that the intention to use will be confirmed by nurses, and the number of nursing problems, such as bladder dysfunction-induced bedwetting, will be reduced with a high rating of DAT usability.

**Conclusions:**

First, this study aims to produce multilevel innovative impacts, including practical, scientific, and societal effects. The results will provide practical solutions for workload reduction in the field of nursing support for continence care, where digital assistive technologies are becoming increasingly important. The DFree ultrasonic sensor is a new technical tool for the treatment of bladder dysfunction. Generating feedback to improve technical applications can increase the user-friendliness and usefulness of the device.

**Trial Registration:**

Deutsches Register Klinischer Studien DRKS00031483; https://drks.de/search/en/trial/DRKS00031483

**International Registered Report Identifier (IRRID):**

PRR1-10.2196/47025

## Introduction

Bladder dysfunction is a health problem that affects approximately 50 million people worldwide [1]. Urinary diseases are diverse and range from simple cystitis to acute postrenal kidney failure [2]. Physical limitations caused by bladder dysfunction can lead to other problems, such as general insecurity, iatrogenic damage, shame, skin damage, irritability and cognition or instability, and the risk of falls [3]. Bladder dysfunction increases the workload of nurses and the costs of care facilities [4-7]. In a rapidly aging society, the number of people with bladder dysfunction will continue to increase [8,9]. “Excretion support,” as part of planned, professional nursing care processes pertaining to the activities of daily living [10,11], benefits from digitization when it improves structure, process, and outcome quality in continence care [7]. New digital assistive technologies (DAT) [12,13] possess great potential for meeting both current and future challenges in continence nursing care [14] as DAT promote independence and participative opportunities for individuals with urological conditions [15,16] and support the care of people with bladder dysfunction [17-20]. As the ability to control bladder muscles represents a milestone in achieving independence in early childhood, the loss of bladder control is psychologically stressful. People affected by bladder disorders often isolate themselves and subsequently experience dramatic reductions in their quality of life [4,21-23]. Besides societal problems such as social isolation, exclusion, and loneliness, bladder dysfunction represents a significant risk factor for more frequent hospitalization and nursing home admissions [24]. For example, an increased risk of hip and thigh fractures was associated with urinary incontinence and nocturia [23]. Despite the availability of an increasing number of DAT to support continence care [6,20], the health systems mostly use wearable absorbent products or absorbent pads to report urine leakage and alarms that are evinced with audible, visual, or graphic signals [4-6]. DFree enables incontinence relief by predicting micturition needs and helping people regain control of micturition [25,26]. However, the relief DFree provides and the willingness of nurses to use DFree remain unclear. Despite the high degree of user acceptance demonstrated within a group of patients in preliminary studies [14,19], the questions of workload reduction in continence care for nurses and cost reduction for continence care materials remain unanswered. To bridge this research gap, this study investigates the use of DFree Professional in an inpatient care setting to extend the results of the previous groundwork. This study examines how the DFree Professional facilitates continence care in the inpatient setting and how health and nursing care professionals assess the “usability” of this DAT. Usability refers to the quality of technology use through human-technology interaction, specifically in relation to nursing professionals and their adoption of DAT in their nursing process planning, and this encompasses several aspects, including significance, transparency, and physical access to sociotechnical components, as well as meaningfulness and effectiveness, all of which should be easily accessible to users [27,28]. Usability in this sense depends “on the significance, transparency, and physical access to sociotechnical constellations” [28] as meaningfulness and effectiveness must be particularly accessible to the users [27,28]. Therefore, the following specific research objectives will be evaluated:

1. As a primary objective, it is assessed how DFree supports and facilitates free professional continence care by nurses in inpatient care settings?
2. Identify and describe changes and barriers in the intention to use (ITU) DFree.
3. As a secondary objective, nurses’ reception of integrating sensors and the associated software into the operational organization of inpatient care processes, as well as cocreative designing of a process that has a high level of acceptance, get surveyed.

## Methods

### Design and Conceptual Framework

**Figure 1:**
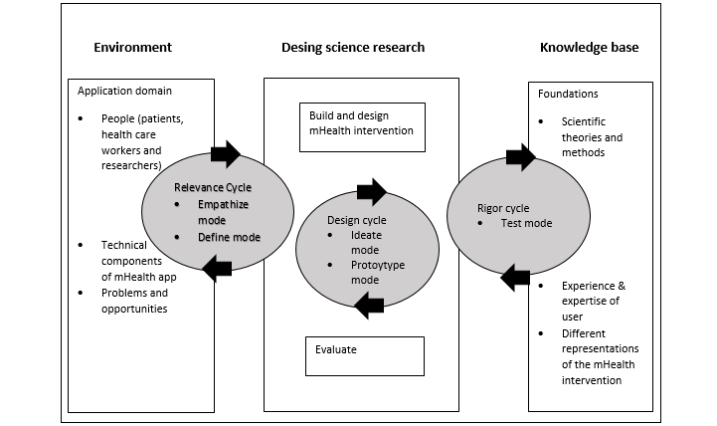
Combination information system research and design thinking (adapted/reproduced from Farao et al [29], which is published under Creative Commons Attribution 4.0 International License [42]). mHealth: mobile health.

To ensure that the DFree achieves high usability and acceptance, this study uses a mixed methods design and follows the methodological specifications of a cocreative user-centered design (UCD) that was previously described by Farao et al [29]. UCD is used to determine the context of mobile health apps and the consequences of their implementation from the design stage [29,30]. The implementation of mobile health apps without end-user involvement compromises desired outcomes and leads to unmet health goals and adverse outcomes [29,31]. UCD is an evidence-based approach that involves end users in the development and implementation of technologies and prioritizes their needs [29,32]. The 2 proven UCD approaches comprise the information systems research framework (ISR) and design thinking (DT). The ISR comprises 3 cycles that integrate the everyday realities of end users (relevance cycle) and the scientific and economic knowledge base (precision and rigor cycles) into the development of technical products (design cycle) [31,33-35]. Furthermore, DT uses different modes that occur in iterative processes; the empathize mode determines the end user’s initial understanding, which is further analyzed in the defined mode to identify specific needs. Subsequently, the development of a technical product undergoes several rounds of ideation (idea mode, ie, generating multiple ideas), prototyping (prototyping mode, ie, translating ideas into physical representations), and testing (testing mode, ie, refining and improving the prototypes and simulating their use in context). In a further step (implementation mode), a medium- to long-term result evaluation is undertaken that possibly leads to the first step for determining need [36]. Despite these advantages, both approaches have disadvantages. The role of stakeholders in ISR is unclear, particularly in the relevance cycle. This means that scientists are primarily responsible for laboratory and field tests and that stakeholders only participate to a small extent in the planning and implementation stages. In the design cycle, the technical requirements are disconnected from feasibility, whereas in the precision cycle, economic interests dominate the users’ points of actual applicability [37]. In DT, the components that improve usability and effectiveness are unclear, and there is no evidence of the generalizability of the identified key needs to a broader group [36]. UCD combines these 2 user-centric approaches and thereby balances the disadvantages of 1 model with the advantages of the other (Figure 1) by combining empathy and the definition mode of DT and integrating the relevance cycle of ISR [29]. Both approaches show positive effects for improving the identified outcomes, higher usability, and user acceptance of technical products [36,37]. Additionally, the combination pursues the purpose of specifying technology requirements with regard to needs assessment as well as improving cooperation between scientists and end users. Thus, intervening in the precision cycle (ISR) with the test mode (DR) connects the knowledge base of the precision cycle with end-user cooperation for product evaluation, as relying only on the test mode is inadequate to enable further technical product development. Instead, the abovementioned combination makes it possible to include health goal attainment as an evaluation factor of product function and success. In particular, with regard to the design cycle mode, the preliminary work of the research group helped to modify the tasks of this project [14]. The design cycle of the ISR is linked to the idea and prototype modes of the DT. With this combination, end users participate in technical (further) development. An advantage of ISR is that the necessary knowledge for further technical development emerges from empirical results, whereas in DT, the knowledge emerges from end users and developers. Thus, the combination of both approaches was suitable for implementation in this study because of the possibility of comparative analysis to improve DFree by factoring in the interests of users and developers, besides increasing usability. Peters and colleagues [38] showed that usability alone does not enable statements on the actual ITU by potential users, as the satisfaction of basic human needs is a decisive factor in “usability” [28,38-41]. In Basic Psychological Need Theory, the more interaction with a system that satisfies basic psychological needs, the greater the technological engagement of end users [41]. Three basic psychological needs describe more proactive, committed, passive, and demotivated human characteristics: competence (ie, feeling capable), connectedness (ie, feeling connected to others), and autonomy (ie, feeling self-determined) [39-41]. Accordingly, the technological influence on end-user well-being can be better understood and evaluated if the abovementioned 3 basic needs are considered in the developmental process [38], as the needs function as mediating variables between a technical product and the end users’ ITU. This means that an increase in autonomy leads to higher engagement. Motivation to use increases with higher competence, whereas increased connectedness increases well-being and ITU. The model assumes that psychological needs consider 5 different levels of analysis: starting with the introduction of technology (adaptation), through interface interaction (interface), dealing with technology-specific tasks (task), technology-supporting behavior (behavior) for the entire life of the end user (life), and the societal level (society). In the METUX model, the basic psychological needs for autonomy, competence, and connectedness positively mediate the design and outcomes of the end-user experience, such as motivation, engagement, and well-being. These variables represent specific, measurable parameters that developers can use to support the fulfillment of basic needs.

This monocentric, exploratory, mixed methods study was planned with multiple measurement times that correspond to the “Evaluation” phase in the Medical Research Council framework [43]. Therefore, a concurrent mixed methods design was used to facilitate the concurrent collection of different forms of data, separate analysis of the data, and final merging of the findings [44,45]. By combining qualitative descriptions of nurses’ experiences with quantitative ratings of nurses’ ITU, we developed more comprehensive knowledge of the influence of DAT DFree in reducing nurse workload in continence care. This study is the first to test and evaluate a DFree ultrasonic sensor in an inpatient care setting with patients and various health care providers (nurses and physicians). In a (cocreative) process for the integration of DFree into the operational organization of the ward’s everyday routine, concrete scenarios are outlined to make DFree usable for practical implementation. Finally, we will collect data on user acceptance while testing the DAT under real-world, everyday ward conditions.

### Technical Description

**Figure 2:**
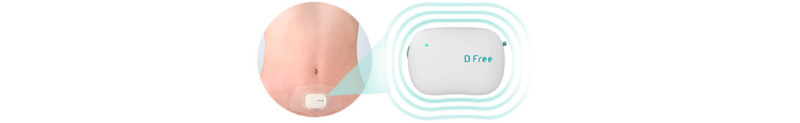
DFree ultrasound sensor.

The DFree Ultrasound Sensor (Triple W Japan KK) is a DAT that comprises 2 parts: a controller and an ultrasound probe that has the potential to predict the need for voiding (Figure 2).

**Figure 3:**
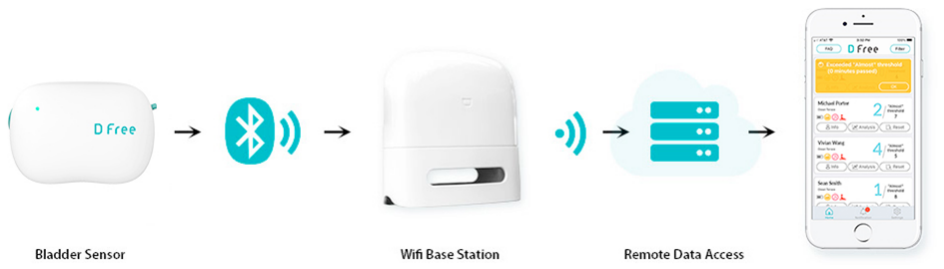
DFree data transfer.

DFree is a supportive tool for patients with bladder dysfunction. The basic principle of the sensor is based on measuring the degree of bladder expansion during filling by using ultrasound measurements, which then notifies nursing professionals through a signal emitted through an iOS operating system app. For this research project, it was necessary to connect the “Wi-Fi Base Station” to the network of the hospital, which comprises internet access (Figure 3). Owing to the strict security measures of university data centers, this interface issue is a specific challenge in the implementation of projects and introducing data analysis technology into health care provisions in general.

DFree uses ultrasound waves at 10-second intervals to measure the degree of change in the urinary bladder during urine filling. A dedicated notebook app provided information on the expected timing of the next visit to the toilet. The display of bladder filling on the screen can be scaled to 10 levels, triggering an alarm when an individually set threshold is reached. The digitally captured degree of bladder expansion was converted into a numerical value and displayed on a mobile phone or notebook app. Thus, DFree represents a potential supportive therapy option for bladder dysfunction that could help nurses reduce their workload.

### Quantitative Evaluation

**Figure 4:**
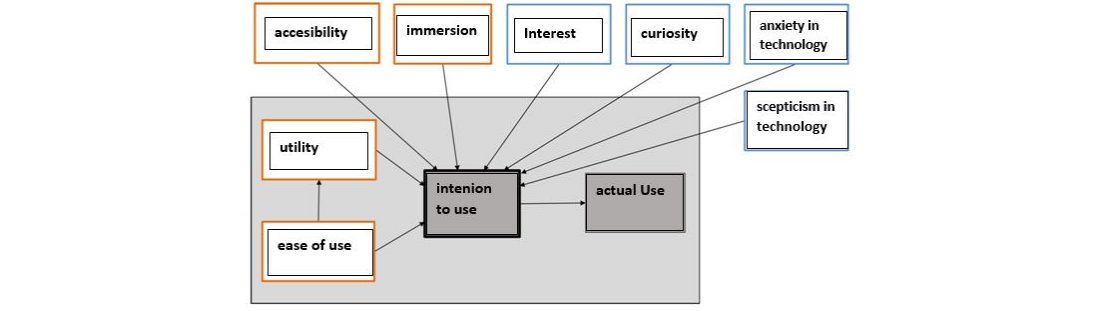
Technology Usage Inventory assessment (adapted from Kothgassner et al [46], with permission from Oswald Kothgassner).

Figure 4 shows technology-specific (orange) and psychological (blue) factors of the Technology Usage Inventory (TUI).

As shown in Figure 4, the 8 TUI scales are divided into technology-specific (orange) and psychological (blue) factors and grouped around the ninth scale, ITU. The wording of the individual items, with the exception of the scales “anxiety in technology” (Technologieängstlichkeit = ANG) and “interest” (Interesse = INT), is related to a specific technology. Each scale comprises 3 to 4 items, each of which is to be rated on a 7-point Likert scale. The INT scale uses 3 items on a visual analog scale to record the ITU’s specific technology. Overall, the TUI consists of 33 items and encapsulates a modular design whereby individual scales are excluded and item formulations must be adapted to the circumstances (eg, specific naming of technology). The internal consistencies of the scales can be rated as good overall (Cronbach α=.70 to α=.89). Furthermore, the TUI scales (with the exception of ZUG) based on heart rate and heart-rate variability have been positively validated as indicators of stress and were positively validated as indicators of stress and relaxation [46]. Except for the immersion (IMM) scale, all the scales offered by the TUI were used in the DFree study. Immersion refers to the evaluation of the intensity of sensory impressions when using a specific virtual reality app, and this scale is excluded because it was not part of the research question (Table 1).

**Table 1:** Description of the Technology Usage Inventory Scales.

Scale	Description
Curiosity (NEU)	Curiosity and inquisitiveness of a person regarding a specific technology
Fear of using a technology (ANG)	Regardless of specific technology. Overwhelm, fear of using technology
Interest (INT)	Regardless of specific technology. Interest in technology and willingness to obtain information independently
User friendliness (BEN)	Perceived user-friendliness (in the sense TAM^a^) of a specific technology
Usefulness (NÜT)	Perceived usefulness (in the sense of TAM) of a specific technology. Refers to support in everyday life.
Skepticism (SKE)	A person’s skepticism and distrust about the use of a specific technology. Assessment of risk, danger, disadvantages
Accessibility (ZUG)	Perceived accessibility (in the sense of availability, buying process) of a specific technology
Immersion (IMM)	Refers to virtual reality apps and is therefore not used here.
Intention to use (ITU)	intention to actually use a specific technology.

^a^TAM: Technology Acceptance Model.

In this study, 3 measurement times (T1, T2, and T3) have been defined (Table 2), and the questionnaire is used at each time point. Before the commencement of the application test (adaptation in the relevance and design cycle), the TUI I Original questionnaire pre-post version—TUI Prä—is used to collect data on a general tendency about technology (fear and curiosity) in the group of nurses before the prototype is introduced. At T2, the prototype is presented for the first time and is thus introduced (design cycle of the interface analysis level). Therefore, the TUI II parallel questionnaire (complete version) will be surveyed in its entirety, and the scales INT, BEN, NÜT, SKE, ZUG, and ITU will be introduced to the nursing staff. At T3 (DT prototyping: testing and implementation mode), measurement will be undertaken, and the group of nursing professionals will test DFree wherein the full version of the TUI II parallel questionnaire (complete version) is represented. The measurement allows statements about changes in the ITU.

For the evaluation, a cumulative value was formed for each scale wherein the ticked answers were added, whereby some items had to be reversed beforehand. The cumulative value starts with 1 as the lowest level of the construct and, depending on the number of items, ranges from 21 (3 items) to 28 (4 items; the highest level). The ITU Scale is an exception because, to evaluate this scale, we measure the distance from the right end point (full rejection) to the answer at the intersection of the line. The distance in millimeters was determined and added to all 3 items (A, B, and C). The maximum scale value achievable is 300. Total scale values are converted into standard values (stanins and percentage rankings) using a standard table (Table 3). Thus, a statement of the relative proportion of participants who achieved the same or lower values is feasible. Therefore, a percentage ranking between 40 and 60 represents the absolute average [46].

The System Usability Scale (SUS) assesses the usability of a system that is subjectively perceived by the user and is demonstrably technology-independent, that is, SUS can be used for a wide range of systems and technologies [47,48]. The 10 items of the SUS are divided into 5 positive and 5 negative statements, each of which is represented on a Likert scale from 0 to 5. The responses are used to derive the SUS item score, which is then converted into the overall SUS score (from 0 to 100). To calculate the overall SUS score, the raw value minus 1 was calculated for all odd items in the first step, whereas the raw value of 5 was subtracted from the even items. For example, if item 1 had a raw score of 4, the score would be 3 (obtained as 4 − 1). For item 2, if the raw score was 2, then the score was 3 (derived as 5 − 2). Next, the newly calculated scores were summed and multiplied by 2.5 to obtain the overall SUS score [49]. Systems are considered fit if they achieve a benchmark of 68 [48,49]. The score is not to be understood as a linear percentage but moves on a percentile and requires a corresponding interpretation [48-50]. In a preliminary study, an adjective scale was developed for a more comprehensive classification of the SUS scores [50] (Table 4).

**Table 2:** Measurement times Technology Usage Inventory (TUI).

Measure times	TUI version^a^	Scale
T1: Adoption—before the technology was introduced	TUI original questionnaire (pre-post version)	NEU^b^ and ANG^c^
T2: Task—testing the technology	TUI II parallel questionnaire (complete version)	NEU, ANG, INT^d^, BEN^e^, NÜT^f^, SKE^g^, ZUG^h^, and ITU^i^
T3: Behavior—end of technology testing	TUI II parallel questionnaire (complete version)	NEU, ANG, INT, BEN, NÜT, SKE, ZUG, and ITU

^a^Designation according to the official TUI manual (Kothgassner et al [45]).

^b^NEU: curiosity.

^c^ANG: fear of using a technology.

^d^INT: interest.

^e^BEN: user friendliness.

^f^NÜT: usefulness.

^g^SKE: skepticism.

^h^ZUG: accessibility.

^i^ITU: intention to use.

**Table 3:** Percentage ranks and stanins.

Percentile rank	Stanine	Percentage (%)
0-4^a^	1	4
>4-11^a^	2	7
>11-23^b^	3	12
>23-40^b^	4	17
>40-60^c^	5	20
>60-77^d^	6	17
>77-89^d^	7	12
>89-96^e^	8	7
>96-100^e^	9	4

^a^Strongly below average.

^b^Slightly below average.

^c^Average.

^d^Slightly above average.

^e^Strongly above average.

The measurement time T1 of the SUS was parallel to the measurement time T3 of the TUI at the end of the trial.

Statistical advice for study planning was obtained from the Institute for Medical Epidemiology, Biometry and Computer Science. As this involved an explorative approach, calculation of the number of cases was unnecessary and was therefore derived from investigations by Faulkner [51], who examined the number of problem identifications in relation to their dependence on the number of subjects. With a sample size of 5 subjects, an average of 85% and at least 55% of 100 problems could be identified, with a very high SD of 9.3. Faulkner [51] indicates that at least 50 subjects are needed to identify an average of 100% of the 100 given problems. As the study duration and the intensity of data acquisition are limited by the human resources available for the project and the project duration, this study used a sample size of up to 45 nurses, which means that 15 nurses are evaluated per ward and per clinical unit.

**Table 4:** Adjective scale of the System Usability Scale (SUS) score (Bangor et al [50]).

Acceptance range and SUS score		Adjective scale
**Acceptable**		
	90-100	Outstanding: best imaginable
	80-89	Excellent
	68-79	Good
**Marginal**		
	50-67	So-so
**Not acceptable**		
	35-49	Mad
	0-34	Very bad: not imaginable

### Qualitative Evaluation

The number of nursing staff members who participate in the expert interviews [52] is limited to 6 to 10 nurses. The selection follows the principles of theoretical sampling, and the aim of the surveys is not to achieve statistical representativeness [53]. Using guided interviews, the survey will be conducted with the clear aim of obtaining specific knowledge that is necessary to answer the precise (and theoretically embedded) research question. The interviews conducted after the completion of the empirical research program on the DFree Professional are useful for obtaining either supply-relevant and “practical” background knowledge or information on the practical implementation of the ultrasonic sensor [52]. The methodological preparation was based on the mechanism-exploring research paradigm of qualitative content analysis that was described by Gläser and Laudel [54], who suggested a 5-step approach commencing with the development of the interview guide and ending by logging the respective surveys [52,54]. Herein, individual steps may be simultaneously taken in parallel to optimize the overall processing time for data collection. The sets of questions fixed in the guidelines were based on preliminary considerations and were developed into a complete guide after the interviews in the first submission (Multimedia Appendix 1).

The methodological recourse to guided interviews for data collection originated from the fact that conducting expert interviews requires good knowledge of the field of investigation [14]. However, challenging hurdles to field access exist within the framework of the DFree study and include the implementation of the DAT and its components in the ecosystem of a clinical ward during normal operational business hours. For example, Adner and Kapoor [55,56] argue that the impact of external challenges through innovation depends not only on their magnitude but also on their position in the respective ecosystem. This implies that the physical functionality of the technology during regular operations in the clinical ward ecosystem is of fundamental importance for assessing the technology itself. Overcoming these hurdles ensures that the surveys of nursing staff on their experiences using DFree Professional within the framework of the nursing process are actually theory-based and methodologically reflected. In the expert interviews, narrative elements relate to the perception of free fitting to problems in continence care from the perspective of nurses are a key focus area [57,58]. The interview guidelines have been developed iteratively using SPSS Statistics 25 [59]. At the end of the 3-month trial period, face-to-face interviews will be conducted using the Phillips Voice Tracer device. All interviews will be transcribed and analyzed based on the content analysis method [54] supported by Microsoft Excel 2016 (Microsoft Corporation) to process data.

All nursing professionals in this study will be recruited through contact with the University Clinic Halle (Saale) and will include those who are employed in clinics for neurology, neurosurgery, and geriatrics. The staff council was informed in advance, and approval of the project was requested.

### Ethics Approval

All procedures involving human participants or human tissue will be performed in accordance with the institutional and national research committee ethical standards and tenets of the 1975 Declaration of Helsinki and its later amendments or comparable ethical standards. Informed consent will be obtained from all the participants. This study was approved by the Ethics Committee of the Faculty of Medicine, Martin-Luther-University Halle-Wittenberg (approval no. 2023–031, dated May 9, 2023). The study was registered in the German Register of Clinical Studies (registration no. DRKS00031483), and the protocol has not been published previously.

## Results

Data collection would begin in the summer of 2023, and the initial results are expected in the winter of 2023. This study will evaluate the implementation of DFree to support nurses in reducing their workload pertaining to continence care. A change in ITU (TUI Scale) is expected. To investigate the possible causes for the measured change, 7 additional characteristics—curiosity (NEU), anxiety about technology, interest (INT), usability (BEN), usefulness (NÜT), skepticism (SKE), and accessibility (ZUG)—will be evaluated 3 times. Furthermore, this study will generate structured feedback that can support developers in further improving DAT DFree. Consequently, a reduction in the number of unsuccessful toilet visits or uncontrolled micturition events while wearing DFree will be recorded and documented by nurses through numerical counting. This will show whether the use of DFree is a practical and advantageous measure for mitigating the nurses’ workload in the context of continence care. As nurses are often poorly equipped to respond to such DAT implementation, the results should serve as a guideline for further improvement of DFree. The combination of qualitative and quantitative instruments addresses the additional aspects that nursing staff would have in mind but will not be assessed using only a quantitative instrument (TUI). Additionally, a change in the perceived usability of the DFree app among nurses is expected.

## Discussion

This pilot study will implement DFree in continence care, give nurses an opportunity to try DFree in real-world practice, and enable the possibility of further cocreative development. The study design enables a novel practical testing of a DAT together with an opportunity for cocreative improvement through direct user feedback. This was the first pilot study to evaluate the use of the DFree ultrasound sensor in an inpatient care setting. This study is the first of its kind and is highly relevant, especially in the context of the barriers to the practical implementation of measures for the digital transformation of health care. The results and experiences from this study facilitate an effect assessment for subsequent studies and serve as a basis for methodological and organizational modifications. Therefore, it is important to evaluate quantitative data in terms of technically meaningful measures. Nonetheless, the study has some limitations, such as the small sample size, diversity of bladder dysfunctions (urinary incontinence and urinary retention), and the impossibility of extrapolating quantitative results to other populations. Additionally, no control group has been included in this study. Nevertheless, this pilot study provides the first assessment of nurses’ ITU a DAT, the DFree ultrasound sensor, and provides initial data for further controlled clinical studies. The strengths of this study include the triangulation of quantitative and qualitative data. With regard to the first question on increasing usability, an increase in the SUS score could be considered a technically meaningful measure. Furthermore, changes in the TUI-ITU scale reflect changes in intended use, which is a logical preliminary stage for actual use of the intervention. Finally, at the societal level, the study’s results will help explain the influence of DFree on reducing the workload of nurses and encourage nurses to use the DAT in nursing care as an additional resource for better outcomes in bladder dysfunction interventions.

In conclusion, this research protocol has been designed to develop a reflective implementation strategy for DAT with a focus on the provision of support in continence care based on scientific and experiential knowledge. The possible use of the intervention is initiated by a change in the ITU as a logical preliminary stage pertaining to actual use. The possibility of practical testing influences the intended use, which, in the next step, affects the actual use in everyday work. The results of this study will encapsulate practical, scientific, and societal implications and would likely pave the way for further studies and interventions to reduce nurses’ workload.

## Appendix

Multimedia Appendix 1 Interview guideline Dfree Trial.

## Abbreviations

ANG: fear of using a technology
BEN: user friendliness
DAT: digital assistive technologies
DT: design thinking
IMM: Immersion
INT: interest
ISR: information systems research framework
ITU: intention to use
NEU: curiosity
NÜT: usefulness
SKE: skepticism
SUS: System Usability Scale
TUI: Technology Usage Inventory
UCD: user-centered design
ZUG: accessibility

## Data Availability

The data sets generated and analyzed in this study are available from the corresponding author upon reasonable request.
