# Supplementary figures and images for: Ultrasound-Assisted Continence Care Support in an Inpatient Care Setting: Protocol for a Pilot Implementation Study
Source: JMIR Res Protoc. 2023 Jul 13;12:e47025. doi: 10.2196/47025 (PMC10375397; doi:10.2196/47025)

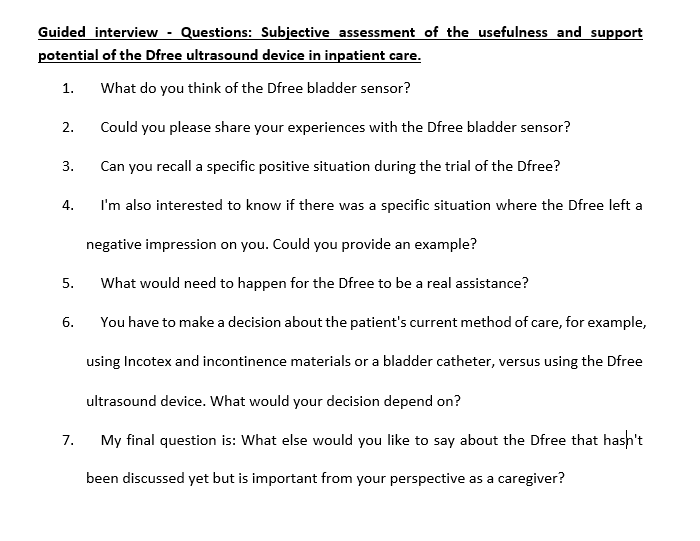

Supplement: Multimedia Appendix 1 [file resprot_v12i1e47025_app1.png]
